# UAV Trajectory Control and Power Optimization for Low-Latency C-V2X Communications in a Federated Learning Environment

**DOI:** 10.3390/s24248186

**Published:** 2024-12-22

**Authors:** Xavier Fernando, Abhishek Gupta

**Affiliations:** Department of Electrical, Computer and Biomedical Engineering, Toronto Metropolitan University, Toronto, ON M5B2K3, Canada; fernando@torontomu.ca

**Keywords:** queuing delay, processing delay, C-V2X, unmanned aerial vehicles, Doppler spread, OTFS, 6G, federated learning, fed-DDPG

## Abstract

Unmanned aerial vehicle (UAV)-enabled vehicular communications in the sixth generation (6G) are characterized by line-of-sight (LoS) and dynamically varying channel conditions. However, the presence of obstacles in the LoS path leads to shadowed fading environments. In UAV-assisted cellular vehicle-to-everything (C-V2X) communication, vehicle and UAV mobility and shadowing adversely impact latency and throughput. Moreover, 6G vehicular communications comprise data-intensive applications such as augmented reality, mixed reality, virtual reality, intelligent transportation, and autonomous vehicles. Since vehicles’ sensors generate immense amount of data, the latency in processing these applications also increases, particularly when the data are not independently identically distributed (non-i.i.d.). Furthermore, when the sensors’ data are heterogeneous in size and distribution, the incoming packets demand substantial computing resources, energy efficiency at the UAV servers and intelligent mechanisms to queue the incoming packets. Due to the limited battery power and coverage range of UAV, the quality of service (QoS) requirements such as coverage rate, UAV flying time, and fairness of vehicle selection are adversely impacted. Controlling the UAV trajectory so that it serves a maximum number of vehicles while maximizing battery power usage is a potential solution to enhance QoS. This paper investigates the system performance and communication disruption between vehicles and UAV due to Doppler effect in the orthogonal time–frequency space (OTFS) modulated channel. Moreover, a low-complexity UAV trajectory prediction and vehicle selection method is proposed using federated learning, which exploits related information from past trajectories. The weighted total energy consumption of a UAV is minimized by jointly optimizing the transmission window ($L_{w}$), transmit power and UAV trajectory considering Doppler spread. The simulation results reveal that the weighted total energy consumption of the OTFS-based system decreases up to 10% when combined with federated learning to locally process the sensor data at the vehicles and communicate the processed local models to the UAV. The weighted total energy consumption of the proposed federated learning algorithm decreases by 10–15% compared with convex optimization, heuristic, and meta-heuristic algorithms.

## 1. Introduction

Sixth-generation (6G) communication networks are increasingly being characterized by non-terrestrial networks (NTNs) that operate using a terrestrial gateway to enable communication between ground nodes and aerial base stations [1]. In unmanned aerial vehicle (UAV)-assisted cellular vehicle-to-everything (C-V2X) networks, UAVs can be used as aerial data centers to store vehicle routes, traffic alerts, and calculate collision probabilities [2]. However, the UAV power consumption varies due to random fluctuations in the vehicular data traffic [3]. Hence, in a transmission window, maximizing the UAV communication range is critical to boost network capacity and improve performance. To improve the data rate and energy efficiency, the UAV trajectory and vehicle data transmission strategies should be optimized jointly [4].

The third-generation partnership project (3GPP) aims to develop 6G technologies and standards that integrate UAVs into terrestrial vehicular communications [5]. The 3GPP Release 16 and beyond include specifications for C-V2X communication that consider the effects of mobility, dynamic channel conditions, and latency challenges [6]. Maintaining robust communication in scenarios with fading channels and frequent link adaptations is a prevailing challenge in UAV-assisted C-V2X communication. If UAV computing resources are intermittently unavailable, it causes interruptions, and the vehicle’s sensor data can become outdated relatively quickly [7]. Furthermore, machine learning techniques are being explored to address C-V2X channel dynamics to address the time-sensitive nature of vehicular data. This is aimed at achieving wider coverage by UAV, as well as providing uninterrupted processing capability to vehicular data [8]. In this paper, the UAV-assisted C-V2X communication channel is modulated using orthogonal time–frequency space (OTFS) modulation to improve performance in scenarios with high mobility, non-line-of-sight (NLoS) conditions and multipath environment [9]. OTFS transforms the wireless channel into the delay-Doppler domain, where the instantaneous channel conditions appear to be stationary in high-mobility scenarios [10]. In the delay-Doppler domain, delay represents the time delay of the signal, and Doppler represents the rate of change of the signal frequency.

**Figure 1:**
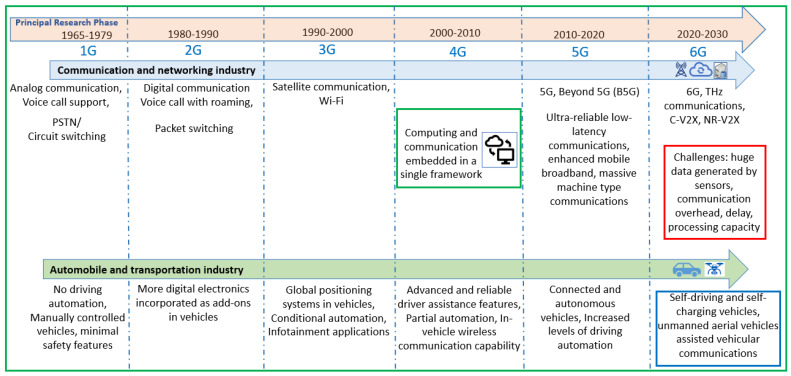
A brief timeline depicting the amalgamation of wireless communication technologies with transportation systems. Also illustrated is the gradual integration of UAVs in vehicular networks in 5G and 6G wireless communication paradigms. A detailed timeline and comprehensive overview of the recent and evolving applications of machine learning techniques in UAV communication frameworks can be found in [14,15].

UAVs can be utilized to improve the coverage range and communications performance of vehicles. Due to the high mobility, UAVs can provide on-demand services to vehicles by adequately overcoming geographical constraints [11]. However, maximizing the UAV available battery power is challenging and comprises a multi-agent, multi-objective, co-operative non-convex optimization problem [12]. Moreover, the UAV-assisted C-V2X environment and the UAV trajectory are typically unknown and time-varying [13]. Therefore, a co-operative UAV-assisted C-V2X communication scenario that maximizes the available battery power of the UAV and optimizes its trajectory while serving the maximum number of vehicles is investigated in this paper. A brief timeline depicting the amalgamation of wireless communication technologies with transportation systems is illustrated in Figure 1. Figure 1 also illustrates the gradual integration of UAVs in vehicular networks in 5G and 6G wireless communication paradigms. Note, each vehicle captures a different kind of data packet, leading to non-independently identically distributed (i.i.d.) and heterogeneous data, requiring significant computational and storage resources. Moreover, a detailed overview and the evolution timeline of the recent applications of machine learning techniques for performance enhancement of UAV communication frameworks can be found in [14,15].

Existing works have investigated federated learning (FL) techniques to effectively improve the quality of service (QoS) in UAV-assisted vehicular communications [16]. Due to the unpredictable trajectory of UAVs and the limited battery power, the fairness of vehicle selection in each transmission window needs to be considered to enhance the real-time coverage rate [17]. Owing to the limited battery power of UAVs, the maximum achievable QoS is limited. Therefore, FL-based mechanisms to ensure an optimal UAV trajectory to maximize the battery power utilization of UAVs must be investigated for low-latency communications between vehicles and UAV [18]. Some recent works have proposed FL to design energy-saving algorithms by jointly optimizing the UAV flight distance and flight height [19]. Maximizing the minimum remaining energy of the UAV is also investigated as a viable solution to extend the UAV battery usage. Federated reinforcement learning (FRL) is also shown to provide superior performance in co-operative UAV–vehicle communications [20]. In certain Markovian approaches, the UAV and vehicles observe their individual states and also learn the state information of other agents to improve the level of co-operation among participating agents. However, obtaining the actions of UAV and other vehicles increases the dimension of the state space [21]. Multi-agent co-operation is also shown to have slow convergence in high-dimensional spaces. For high-dimensional spaces and multi-agent cooperation, the learning efficiency can be improved using FL-based optimization algorithms that assist the participating agents in faster decision-making [22].

A UAV must achieve wider ground coverage due to line-of-sight (LoS) channel links from an optimum UAV altitude [23]. However, in a multi-vehicle network with spatio-temporal correlation in transmitted data, the UAV energy constraints, flight trajectory restrictions, and QoS requirements need to be jointly considered [24]. Because of the multipath propagation effects, vehicles encounter non-line-of-sight (NLoS) links, and the channel quality and transmission rate deteriorate. The signal frequency varies due to the relative motion between the UAV and the vehicles [9]. The UAV trajectory must be optimized considering the constraints of the data rates and bit error rate (BER), taking into account the Doppler spread [10]. Accumulating and processing data from different transmission time intervals (TTIs) leads to delay [25].

### 1.1. Contributions

This paper investigates energy-efficient UAV trajectory optimization and computing resource allocation in UAV-assisted C-V2X communication and is a significant extension of our previous work in [26]. This paper also extends our previous work in [27], where we investigated some robust and strategic game-theoretic approaches used by the UAV to select different vehicles in each TTI. The main contributions of this paper are as follows:

1. We propose to examine end-to-end packet latency as well as UAV energy consumption based on FL with varying numbers of vehicles. From the FL iterations, we study the probability of the optimal trajectory prediction of the UAV using different neural network models. This is a significant extension of our previous work in [28] where we analyzed the variation in the UAV transmit power for a varying number of vehicles in a gross data offloading scenario.
2. As a function of computation offloading to UAV and the local vehicle model computation time, we plot the average task completion latency for varying numbers of vehicles. Using long short-term memory (LSTM), gated recurrent unit (GRU), recurrent neural network (RNN) and convolutional neural network (CNN)-LSTM models, we compare the average task completion latency for gross data offloading and FL, and the probability of optimal UAV trajectory prediction.
3. We validate the proposed solution by calculating the number of training iterations required to satisfy the service-time constraint for LSTM, GRU, RNN, and CNN-LSTM models. Here, we use the *V2X-Sim* and *LTE I/Q* datasets and based on the number of vehicles that exceed a specified time frame to process a task, we conclude the maximum number of vehicles a UAV can support without violating the identified constraints. Furthermore, we utilize the *V2X-Sim* dataset to verify the FL model convergence characteristics and performance trade-offs [29] for the proposed UAV-assisted C-V2X communications.
4. Unlike existing works where the device-to-device communication largely depends on neighbor discovery [30], in this work, the TTI is selected using the distributed scheduling protocol known as sensing-based semi-persistent scheduling (SPS) [31]. Since the vehicles and the UAV operate at different speeds, SPS is utilized to enable vehicles to independently select and manage the available bandwidth and the UAV communication and computational resources.

### 1.2. Organization

The remainder of this paper is organized as follows. Section 2 discusses some of the recent literature that applied FL and FRL techniques to achieve performance improvement in UAV-assisted vehicular communications. The section also identifies the challenges and opportunities for the performance enhancement of UAV-assisted C-V2X communications using FRL [8]. Section 3 illustrates our system model and discusses the UAV vehicle communication architecture used in this work. Section 4 presents our problem formulation where we formulate the problem of latency minimization, power optimization, and UAV trajectory control in UAV-assisted C-V2X as a Markov decision process (MDP). Here, a mixed-integer non-convex UAV trajectory optimization problem is proposed and is divided into four optimization sub-problems using Lagrangian dual decomposition. Section 5 outlines our proposed proposed solution approach, where Q-learning and policy gradient learning are applied at each vehicle to generate the local model parameters. Here, federated-DDPG using the LSTM model with temporal aggregation is proposed to enhance the UAV trajectory prediction. Additionally, federated reinforcement learning (FRL) is investigated to minimize the UAV energy consumption for time-varying data size and channel conditions. Section 6 discusses the findings of this work and investigates the impact of a control parameter on optimal UAV trajectory prediction. Here, we also compare the latency observed for packets of varying byte sizes for a varying number of vehicles and available UAV power. Section 7 concludes the paper and discusses some avenues for future research. The main abbreviations used in this paper are described in Table 1.

## 2. Related Work

Some novel applications of UAVs in various domains are listed in [32]. Recently, UAVs have found applications in monitoring the stability of landfills, monitoring the frequency of settlements, and, consequently, safeguarding the people living near landfills from serious hazards [33]. Some studies have investigated the applicability of UAV-based photogrammetry to monitor geometric changes in landfills to provide precise information to make informed reclamation decisions [33]. Other works have proposed UAV-based antennas and propagation measurements for electromagnetic field assessments, as they are flexible, cost effective, and easy to deploy [34]. The authors in [34] also evaluated some channel models for UAV positioning accuracy and antenna alignment in the presence of large-scale antenna arrays. The authors reported that some inaccuracies in positioning were alleviated by virtue of the recent advances in portable measurement systems and antennas designed specifically for UAVs [34]. Moreover, some authors have hypothesized that as the UAV-assisted wireless channels are overt, the UAV communications are vulnerable to eavesdropping attacks [35]. The authors in [35] proposed novel solutions to enhance UAV communications’ minimum secrecy rates. The authors formulated an optimization problem with parameters such as user association variables, UAV trajectory, and output power and proposed a solution based on sequential decision-making. Specifically, the work proposed and utilized a single agent soft actor critic and twin delayed deep deterministic policy gradient algorithm to jointly optimize the aforementioned parameters [35]. However, jointly optimizing the UAV trajectory and power consumption was not addressed by these works. In some recent works, it has been hypothesized that the UAVs do not operate accurately in indoor environments where the localization performance of global navigation satellite system (GNSS) is suboptimal [36]. This makes it even more difficult to accurately localize UAVs in dynamic environments with weak signal strengths in mission-critical applications and where channel interference is not negligible; hence, some alternatives to GNSS-based positioning were explored in [36]. These solutions estimate the distance and position using the received signal strength indicator (RSSI) based on Bluetooth low-energy beacons.

The authors in [37] proposed a single-agent deep reinforcement learning approach to solve scheduling requests, and a short-term memory was constructed to forecast the traffic. Using echo state networks, a machine learning framework was proposed to predict packet distribution and traffic patterns to optimize UAV flight paths and cached content. The energy consumption of the hybrid online offload framework was minimized by using a deep learning-based hybrid memory to store offloading decisions [38]. In order to implement optimization methods, it was necessary to have complete information about how vehicles distribute their data and how traffic is distributed.

The authors in [39] analyzed UAV placement strategies to maximize the number of vehicles under coverage to maximize the sum of channel gains or minimize non-convex path loss functions. The Dinkelbach algorithm and successive convex approximation (SCA) were used to maximize the UAV’s energy efficiency by optimizing its trajectory, transmit power, and computational load distribution. In order to minimize overall energy consumption, the authors optimized flight trajectory, transmission power, time slot scheduling, and task data assignment using Lagrangian duality. To optimize both the UAV position and its computing resources simultaneously, a three-stage iterative method was proposed. Based on ideal LoS channels, independent of multipath channels and Doppler spread, a mobile edge computing (MEC) network was designed with access to base stations (BSs) and UAVs [40]. Moreover, in the existing works, the UAV-assisted C-V2X communications can be modulated using non-orthogonal multiple access (NOMA), orthogonal frequency division multiplexing (OFDM), or OTFS modulation schemes.

- In NOMA, multiple vehicles share the same time and frequency resources, and their signals are distinguished by different power levels. Vehicles with stronger channel conditions are assigned lower power, while vehicles with weaker channel conditions are assigned higher power [41]. This leads to the efficient utilization of the available resources and enhances the throughput [42].
- OFDM divides the spectrum into multiple orthogonal subcarriers in the frequency domain and uses them to transmit data simultaneously. However, OFDM faces challenges in high-mobility environments, where Doppler spread is significant and the performance degrades in NLoS conditions and multipath environments [43].
- OTFS modulation improves communication performance in scenarios with high mobility, NLoS conditions, and multipath environments [9]. OTFS transforms the wireless channel into a new domain called the delay-Doppler domain, where the instantaneous channel conditions appear to be stationary in high-mobility scenarios [10].

A multi-hop UAV-assisted relay network was employed to facilitate communication between transceivers on the ground by optimizing the channel assignment and flight control of the UAVs [44]. To maximize computation capability, the UAV deployment coordinates and altitude, transmit power, and bandwidth allocation were optimized together with the resource allocation and deployment strategy of the UAVs. By optimizing the communication scheduling, the UAV trajectory, and the computing resources jointly, subject to mobility, connection, and computation constraints, the authors proved that it could lower the signal-to-noise ratio (SNR) based on the first-order Gauss–Markov process and maximize computation capability [45]. An orthogonal frequency division multiple access (OFDMA) multi-user MEC with resource allocation was studied in [46]. A low-complexity suboptimal algorithm was proposed to extract the probable correlations between trajectory alterations and operations that are difficult due to their complexity. To minimize the distance between vehicles, a DRL algorithm using actor–critic was proposed for the stochastic scheduling of MECs [47].

The authors in [10] demonstrated that due to the high-speed movement of UAVs, the estimation and equalization of wireless channels for over-the-air communication are complex tasks. However, it is possible to directly modulate data in the time-delay-Doppler (TDD) domain over a wide range of time frequencies in a multipath propagation channel using OTFS. In high-speed mobile communication systems, OTFS achieves greater diversity gain by adapting to time-varying channels and converting the multipath channel into the TDD domain [48]. Moreover, NLoS signal reflections and multipath propagation cause challenging channel conditions to achieve BER performance for high-spectral-efficiency signals. It was determined that the BER varied depending on the severity of the Doppler spread, SNR, and noise and interference in the channel. Moreover, the choice of modulation scheme within the OTFS framework was also a critical factor [49].

To maximize the network throughput, conventional DRL implementations rely on centralized data collection at the MEC server, which adapts UAV trajectories jointly during each time step. In order to adapt the UAV trajectory, the vehicle scheduling, and energy harvesting policies, a DQN approach was proposed to overcome the excessive communication and training overhead [50]. It was proposed to use the multi-agent DQN method to optimize the real-time downlink capacity for all vehicles, as well as the MADDPG method to assign targets and plan the trajectory of multiple UAVs to gather sensor data. The UAV speed control was adjusted according to its energy status and position using the Q-learning algorithm. Other approaches employed an actor–critic DDPG for jointly optimizing the UAV trajectories and transmission scheduling strategy using the k-means clustering to aggregate different vehicles. In order to minimize the age of information (AoI), actor–critic DRL was used to adapt the sensing and flying decisions of each UAV, and to optimize the UAV–vehicle association strategy and minimize the total flight time [51].

Using DRL in applications with random task arrivals to optimize resource distribution, many approaches have proposed a dynamic scheduling strategy. Using the FL algorithm, collaborative model training can be accomplished without sharing data [52]. In this model, the spatial correlation of the traffic of adjacent vehicles is extracted from the wireless traffic data as images. In order to improve the prediction accuracy, this model employs temporal aggregation to capture the relationship between wireless traffic during current time slots and traffic during upcoming time slots. To reduce the power consumption of the UAV, select multiple starting positions at random to mitigate the impact of the initial positions [53].

In recent machine learning approaches, spatio-temporal neural networks have been used for accurate cellular traffic forecasting, and variations combine the output with past statistical data in order to improve long-term prediction accuracy [54]. An LSTM-based architecture for cellular traffic prediction combines Gaussian process regression with LSTM to capture spatial and temporal dependencies. For extracting dominant periodic components, LSTM is used for learning the long-term relationships among small random values, and FL is applied to estimate the residual random values [55]. The FL methods were shown to have lower computational complexity than probability models to predict the baseline component and maximum likelihood estimation methods to estimate the residual component. To reduce the communication overhead for energy saving, graph partitioning and rejoining is utilized in some of the proposed schemes, whereas to predict throughput and minimizing power consumption, LSTM has been used [56].

Recent works have shown that UAV trajectory optimization requires free space pathloss channel model when the UAV is flying at a sufficiently high altitude where the LoS is dominant [57]. However, in urban scenarios, buildings and small-scale fading cannot be ignored, and the channel is characterized by the Rician fading model. To address random channel capacity in Rician fading, some works have proposed to approximate the maximum allowed transmission rate, based on which a trajectory design method is presented to minimize the task completion time in Rician shadowed fading channel models [58]. In some approaches, the ratio of LoS-to-NLoS components and the degree of LoS shadowing is determined. Some approaches involved time slots where the UAV position is assumed to be invariant in each slot, optimizing them to approximate the optimal trajectory [59]. The path loss between a UAV and a vehicle depends on their respective positions as well as the propagation environment, which varies when a UAV is in a rural, suburban, urban, or high-rise environment. The movement of UAVs affects the amplitude, phase, and delay of the received signal [60]. Some advantages of FRL in latency minimization of UAV-assisted C-V2X networks are summarized in Table 2.

## 3. System Model

**Figure 2:**
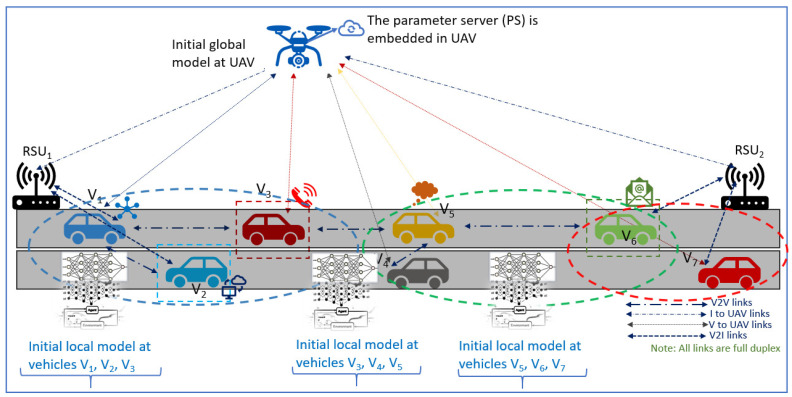
System Model: Delay is accumulated as vehicles in different clusters generate and transmit local models to the UAV. The UAV transmits the global model to the vehicles. Note, each vehicle captures a different kind of data packet, leading to non-i.i.d. and heterogeneous data.

The system model considered in this paper is illustrated in Figure 2. In a time period (T), the UAV has available battery power ($P_{b}$), flying at a height (H) in meters (m). The time period (T) is divided into multiple transmission windows ($L_{w}$), where *V* vehicles sense and process sensor data and transmit the processed local models to the UAV.

As per Equation (1), the data rate in bits/Hz transmitted by each vehicle over UAV flight time is computed based on the summation of the instantaneous transmission rate ($s_{i,t}$):

(1) $$s_{i,t}\;\left(\psi_{t},\;w_{t},\;D_{t}\right)\;=\;B_{j}\;\log_{2}\;\left(\;1\;+\;\frac{P_{i}^{t}{\left|h_{i,j}^{t}\right|}^{2}}{\sum_{j=1}^{T-1}\;P_{{\bar{d}}_{j}}{\left|h_{i,j}^{t}\right|}^{2}\;+\;B_{j}{\left(\sigma_{i,j}^{t}\right)}^{2}}\;\;\right)\;$$

where $\psi_{t}$ is a binary variable, indicating whether a vehicle transmits local models to the UAV in a transmission window ($L_{w}$) or not, $w_{t}$ is the FL model weights at time *t*, and $D_{t}$ is the delay, which is the sum of the queuing delay and processing delay.

### 3.1. Packet Arrival at the UAV

The bandwidth is denoted by $B_{j}$, and $P_{i}^{t}$ indicates the power consumed by the UAV while communicating with the *i*th vehicle. The term $\sigma^{2}$ is the noise power density at the receiver of the vehicle. The channel gains of the UAV at time slot *t* are denoted as $h_{i,j}\left[t\right]$. The communication links can be LoS or NLoS with probabilistic path loss depending on the UAV position, obstacle height, number of obstacles, and the UAV height (H). The instantaneous transmission rate for the *i*th vehicle in a time slot is given by Equation (2) as

(2) $$s_{j}^{i}\left(b_{j}^{i},x_{i},y_{i}\right)=b_{j}^{i}\log_{2}\left(1+\Gamma_{j,i}\right)$$

where $b_{j}^{i}$ is the instantaneous bandwidth utilized by the *i*th vehicle in the *j*th transmission window, and Γ is the SNR. The packets comprising processed local models arrive at the M/M/k queue where the inter-arrival time ($\lambda_{t}$) between successive packets is exponentially distributed, and in each TTI, an arrival is independent of the previous arrivals. The local models ($\Psi_{j}$) considered for federated averaging, similar to the approach proposed in [26], are the summation of local models from all vehicles (∀i∈V) transmitted in the previous TTIs as given by Equation (3):

(3) $$\Psi_{j}\left(b_{j}^{i},x_{i},y_{i}\right)=\lambda_{t}\sum_{t=1}^{T}\psi_{j}^{i},\;\forall i\in V$$

The local models arrive at the queue, which accumulates at a rate specified by Equation (4). For uplink transmission, the data transmitted from a vehicle at time *t* are restricted by the available bandwidth ($B_{i}^{t}$) and uplink transmission window ($\tau_{u}$):

(4) $$\psi_{j}^{i}=\left\{\begin{array}{cc} s_{j}^{i}\left(b_{j}^{i},x_{i},y_{i}\right), & \mathrm{if}\;\tau_{i}\leq i\leq \lambda_{i}\\ \min(B_{i}^{t},s_{i}^{t}\times \tau_{u}), & \mathrm{otherwise} \end{array}\right.$$

### 3.2. UAV Power Consumption

The total power consumption during time period *t* is given by Equation (5):

(5) $$P_{total}\left(t\right)=\sum_{i=1}^{V}P_{i}\left(t\right)+P_{UAV}^{\left(V\right)}\left(t\right)$$

where $P_{UAV}^{\left(V\right)}$ is the power required for operating the UAV during time period *t*, and $P_{i}\left(t\right)$ denotes the power consumption in the *i*th time slot when *V* vehicles transmit data or local models to the UAV. The variation in UAV power $P_{i}\left(t\right)$ is modeled in Equation (6), which depends on the uplink power, downlink power, processing power, and flying power:

(6) $$P_{i}\left(t\right)=u_{i}\left(t\right)\left(\sum_{k=1}^{k}\gamma_{k}\psi_{i,k}\left(t\right)+P_{UAV}^{\left(1\right)}\left(t\right)\right)$$

where $u_{i}\left(t\right)$ is a binary variable; $u_{i}\left(t\right)$ = 1 indicates that in the *i*th time slot, the UAV is processing the data from a vehicle, and $u_{i}\left(t\right)$ = 0 indicates that the UAV is idle which leads to the wastage of energy. Moreover, $\psi_{i,k}\left(t\right)$ denotes the size of the *k*th type of vehicular data served by the UAV in the *i*th time slot. In addition, the coefficient $\gamma_{k}\psi_{i,k}\left(t\right)$ determines the power consumption during the uplink and downlink of the *k*th type of vehicular data, which can either be a local model for different sensor data, basic safety message (BSM), or co-operative perception message (CPM). This paper aims to minimize the weighted total energy consumption by jointly optimizing the transmission window ($L_{w}$), transmit power ($P_{i}\left(t\right)$), and UAV trajectory ($q_{\left(x,y\right)}$) while considering the Doppler spread. The UAV energy consumption depends on its flying speed, flying duration, flying time, and communication with vehicles [68]. The trajectory coordinates traversed by UAV are denoted by $q_{\left(x,y\right)}$ with the flying time upper bound by T. The trajectory coordinates including all flying and turning points are represented as $q_{\left(x,y\right)}\in \left[q_{\left(0,0\right)},q_{\left(0,1\right)}\;\ldots \;q_{\left(1,0\right)},q_{\left(1,1\right)}\;\ldots \;q_{\left(m,m\right)}\right]$.

### 3.3. Distance Between Vehicles and UAV

To calculate the real-time distance between the vehicles and the UAV, we use the Euclidean coordinate system, where the vehicles remain on the same two-dimensional horizontal plane, implying H = 0. The maximum speed of UAV is denoted as $v_{max}$ in meter/second (m/s). When the UAV flies and collects sensing data from vehicles, it must meet the minimum SNR for reliable data collection by spatially distributing the vehicles along the ground. This is challenging in NLoS channel conditions, as the vehicles frequently move in and out of the direct communication range of the UAV. The collected data are buffered at the queue or forwarded to the UAV. Hence, we utilize the *LTE I/Q* dataset for five different UAV altitudes to construct a predetermined 3D map of spatial coordinates collected as the trajectory changes rapidly and abruptly [69]. The distance between the UAV and the *i*th vehicle is given by Equation (7):

(7) $$d_{veh}^{uav}=\sqrt{{\left(x_{u}\;-\;x_{i}\right)}^{2}\;+\;{\left(y_{u}\;-\;y_{i}\right)}^{2}\;+\;H^{2}},\;i=1,2,\ldots V$$

At a specific height (H), the UAV distance traveled in one time slot is constrained by Equation (8) as follows:

(8) $${\left(x_{i+1}-x_{i}\right)}^{2}+{\left(y_{i+1}-y_{i}\right)}^{2}\;\leq \;{\left(v_{max}\delta_{t}\right)}^{2},i=1,\ldots ,V$$

where $\delta_{t}$ is a limiting parameter.

### 3.4. Channel State and UAV Energy Consumption

The orthogonal transmission is employed in the uplink to allow multiple vehicles to simultaneously upload their data to the UAV. The building heights are modeled using Rayleigh distribution. To model the channel state and UAV-to-vehicle links, we consider Rician fading. The LoS and NLoS links have different probabilities of occurrence, which is a function of the environment, density and height of buildings, and the elevation angle between the UAV and vehicles. The geometrical statistics of various environments are offered by the International Telecommunication Union (ITU-R), which determine the density, number, and height of the buildings and other obstacles [48]. The NLoS effect decreases as the elevation angle between the receiver and transmitter increases, and the communication link approaches near LoS. The Rician K-factor that represents the strength of LoS component is a function of the elevation angle and the UAV altitude [49]. The Rician K-factor impacts the maximum transmit power and path loss exponents as given by Equation (9):

(9) $${\hat{h}}_{j}^{i}=\left(\sqrt{\frac{K}{1+K}}\bar{h}+\sqrt{\frac{1}{1+K}}\tilde{h}\right)$$

where ${\hat{h}}_{j}^{i}$ is the channel-gain experienced by the *i*th vehicle in the *j*th transmission window considering the Rician K-factor. The term $\bar{h}$ implies the LoS component, and the term $\tilde{h}$ indicates the fading component.

## 4. Problem Formulation

We formulate an optimization problem to determine the optimal trajectory of UAV in C-V2X communication, where the UAV maximizes the number of served vehicles adhering to QoS and power constraints. In a transmission window ($L_{w}$), we maximize the UAV utility considering the priority of data transmission and link connection time, as well as minimizing the UAV energy consumption formulated as a bin packing problem. We consider the uplink and downlink capacities in vehicle edge servers for transmission rate maximization, energy minimization, and delay minimization. Due to the spatial and temporal correlations between the UAV energy and trajectory, the proposed optimization is a non-convex problem. The objective function and constraints are non-convex with respect to the backhaul link outages and transmission latency, leading to a mixed-integer non-convex optimization problem. Hence, the problem (P1) of energy-efficient computing resource allocation in UAV is formulated as follows in Equation (10):

(10) $$\begin{array}{cc} P1: & \min_{\psi^{\left(t\right)},w^{\left(t\right)},D^{\left(t\right)},T}\{w_{1}\underset{SP_{1}}{\underset{︸}{\left[T\tau_{u}+\sum_{t=1}^{T}\min\left(\max_{v\in V}\left(d_{v}^{t}\right),\tau_{d}\right)\right]}}+\\ & w_{2}\underset{SP_{2}}{\underset{︸}{\left[\sum_{t=1}^{T}\sum_{v\in V}D_{v}^{\left(t\right)}\min\left(d_{v}^{\left(t\right)},\tau_{d}\right)\right]}}+w_{3}\underset{SP_{3}}{\underset{︸}{\left[\sum_{t=1}^{T}\sum_{v\in V}I_{v}^{\left(t\right)}\right]\}}}+\\ & \min_{\Psi ,\Phi ,D_{i},T}\underset{SP_{4}}{\underset{︸}{\sum_{i=1}^{V}\sum_{t=1}^{T}\left(e_{i}\left(t\right)+\sum_{j\in V}\sum_{k\in K}t_{i}p_{i,j}^{\left(k\right)}\varsigma_{i,j}^{\left(k\right)}\left(t\right)\right)}}\\ & \mathrm{subject}\;\mathrm{to} \end{array}$$

(11) $$\begin{array}{cc} C1:\; & \sum_{t=1}^{T}\left(1-I_{v}^{\left(t\right)}\right)D_{v}^{\left(t\right)}=B_{v}^{\left(t\right)},\forall v\in V\; \end{array}$$

(12) $$\begin{array}{cc} C2:\; & D_{v}^{\left(t\right)}\in \left[D_{min},D_{max}\right],\forall v\in V,\forall t\in T\; \end{array}$$

(13) $$\begin{array}{cc} C3:\; & D_{i}\left(t\right)\leq D_{max}\;\mathrm{and}\;D_{i}\left(T\right)=0\; \end{array}$$

(14) $$\begin{array}{cc} C4:\; & \psi_{v}^{\left(t\right)}\in \left\{0,1\right\},\forall n\in V,\forall t\in T\; \end{array}$$

(15) $$\begin{array}{cc} C5:\; & w_{n}^{\left(t\right)}\in \left\{1,\ldots ,V\right\},\forall v\in V,\forall t\in T\; \end{array}$$

(16) $$\begin{array}{cc} C6:\; & \left|\right|q_{i}\left(t+1\right)-q_{i}\left(t\right)\left|\right|\leq v_{max}\left(t\right)t_{i,i+1}\; \end{array}$$

(17) $$\begin{array}{cc} C7:\; & \left|\right|q_{i}\left(t+1\right)-q_{i}\left(t\right)\left|\right|\geq d_{min}\; \end{array}$$

(18) $$\begin{array}{cc} C8:\; & q_{\left(x,y\right)}=q_{\left(x_{0},y_{0}\right)}\cdot e^{-\alpha \left(\frac{x^{2}}{a^{2}}+\frac{y^{2}}{b^{2}}\right)}+H \end{array}$$

(19) $$\begin{array}{cc} C9:\; & w_{max}\left(0\right)=D_{m}\;\mathrm{and}\;w_{max}\left(T\right)=0 \end{array}$$

(20) $$\begin{array}{cc} C10:\; & \varsigma_{i,j}^{\left(k\right)}\left(t\right)\in \left\{0,1\right\}\;\mathrm{and}\;s_{i,m}\left(t\right)\in \left\{0,1\right\} \end{array}$$

(21) $$\begin{array}{cc} C11:\; & s_{i}\left(b_{i}^{\left(n\right)},x^{\left(n\right)},y^{\left(n\right)}\right)\geq \varrho_{i}s_{i}^{min},\;\forall n,i\in I \end{array}$$

(22) $$\begin{array}{cc} C12:\; & 0\leq b_{i}^{\left(n\right)}\leq \varrho_{i},\;\forall n,i\in V\; \end{array}$$

(23) $$\begin{array}{cc} C13:\; & D_{n}\left(\left\{q\left(t\right)\right\},\left\{a\left(t\right)\right\}\right)=\int_{0}^{T}a_{k}\left(t\right)s_{n,\max}\left(q\left(t\right)\right)dt\; \end{array}$$

(24) $$\begin{array}{cc} C14:\; & a_{k}\left(t\right)=\left\{0,1\right\},\forall v\in V,t\in T\; \end{array}$$

where P1 is a multi-objective optimization problem. The constraint C1 is an indicator of the status of a TTI. It is identified as a binary value, 0 or 1, implying whether, in the *i*th TTI, the UAV is communicating with a vehicle or is idle. The constraints C2 and C3 imply that a vehicle is served by the UAV in the current $L_{w}$ subject to an upper bound on delay given by $D_{max}$. The constraints C4 and C5 impose an upper bound on the size of the packets (ψ) and model weights transmitted from a vehicle to the UAV. This ensures that the queue at the UAV is not overloaded and imply that the UAV computing resources consumed do not exceed the maximum baseband processing capacity.

The constraints C6 and C7 restrict the UAV trajectory to ensure that the UAV is not out of the vehicle’s coverage range for a long time and serves at least one vehicle in each TTI. The UAV trajectory is constrained by its initial position and its final position to avoid obstacles, maintain speed and altitude, minimize energy consumption, and maximize travel time. In constraint C8, we bound the UAV trajectory by an elliptical path. The UAV can theoretically move from point $A_{\left(x_{A},y_{A}\right)}$ to $B_{\left(x_{B},y_{B}\right)}$ through infinite possible paths. The constraint C8 represents a Poisson point process within an elliptical area and characterizes the spatial distribution of paths traversed by the UAV. The constraint C9 ensures that the vehicles’ data are successfully offloaded to the UAV during a TTI and UAV flight time (T). The constraint C10 denotes the assignment coefficient for vehicles, where ς = 1 denotes vehicle is scheduled for transmission, while ς = 0 denotes that it is waiting. The constraint C11 introduces a control parameter $\varrho_{i}$ for the data rates to reduce the computational complexity of the solution. The constraint C12 limits the available bandwidth so that all vehicles have a chance to send the maximum amount of data to the UAV. The constraints C13 and C14 indicate the throughput of the vehicle local models. These constraints guarantee that each vehicle uploads the minimum amount of data to the UAV and prevents the UAV from wasting radio resources on a vehicle that cannot be served in a given TTI. The main symbols used in this paper are described in Table 3.

## 5. Proposed Solution

**Figure 3:**
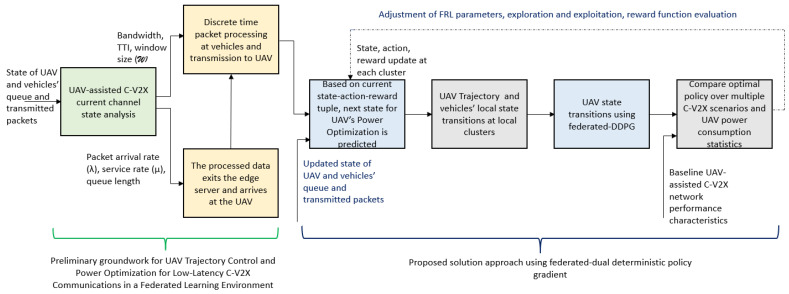
An illustration of the proposed federated reinforcement learning-based solution approach for UAV trajectory control and power optimization for low-latency C-V2X communications.

The problem P1 is divided into four sub-problems pertaining to communication scheduling as well as trajectory and computing resource optimization using Lagrangian dual decomposition. The communication delay is optimized in the first sub-problem $SP_{1}$. Branch and bound is used to solve optimization sub-problems $SP_{1}$ and $SP_{2}$ to find an optimal solution from a set of candidate solutions. Branch and bound is used to allocate limited UAV resources to vehicles to minimize the objective function. For a given delay, the trajectory and computing resource are jointly optimized in $SP_{2}$. Next, we apply binary relaxation to transform P1 into a linear programming problem. Binary relaxation transforms the sub-problems into convex problems, where the complex variables are relaxed into real variables. In sub-problems $SP_{3}$ and $SP_{4}$, the objective function to maximize the data rate, energy efficiency, and the constraints are non-convex and are solved using successive convex approximation (SCA). To integrate the solutions from the four sub-problems with equality and inequality constraints, we use successive quadratic programming (SQP), which is an iterative approximation of the nonlinear objective function and constraints using quadratic models, updating them in each iteration. Figure 3 illustrates the proposed FRL-based solution approach for UAV trajectory control and power optimization for low-latency C-V2X communications.

### 5.1. Long Short-Term Memory to Approximate the UAV Trajectory Parameters

We propose an LSTM to approximate the complex nonlinear functions in the above convex optimization sub-problems. As the LSTM approximates the parameters of the objective function and the constraints, the resulting model is integrated with the optimization framework. In P1, parameters such as the UAV trajectory ($q_{\left(x,y\right)}$) in the next TTI or the vehicle data rate $s_{i,t}$ cannot be precisely known, and vary significantly with time. LSTM is used to learn these parameters from training data, and the learned parameters are incorporated in the solutions of the optimization sub-problems ($SP_{1}$–$SP_{4}$). As the UAV trajectory changes, these parameters change, and as there are temporal dependencies, LSTM models these dynamics. The optimization problem is solved in an iterative manner, where the LSTM adapts the optimization strategy based on the updated parameters. In addition, the LSTM models the time-varying constraints or complex relationships between variables in P1 and predicts constraint violations, as well as adapting to changing constraints. The problem P1 is addressed using the actor–critic framework using two sets of LSTM networks to approximate the policy function ($Q^{\pi }(s_{t},\pi \left(s_{t}|\psi \right)$) and value functions (V(ψ)). The parameterized actor network generates a deterministic action to maximize the value function based on the steady-state distribution of the actors’ policy.

**Figure 4:**
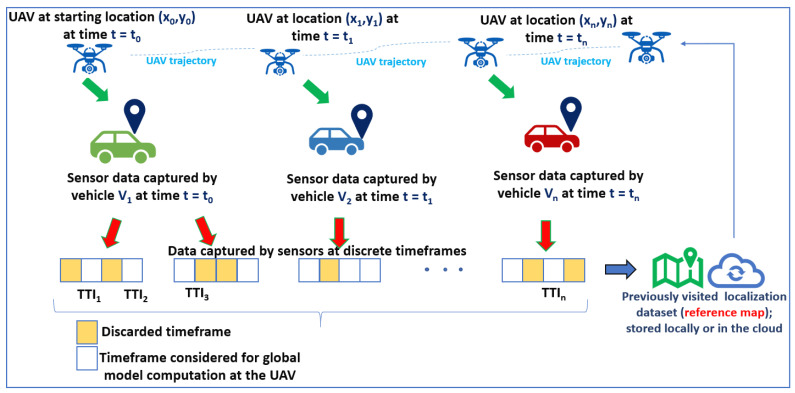
UAV trajectory varies in a random manner, and the vehicles capture varying sensor data at different TTIs. By processing the sensor data, local models are generated at the vehicles and a global model is generated at the UAV.

The critic network approximates the value function by taking the derivative of V(ψ), given by ∇V(ψ) with respect to the policy parameter and updates the actor network by gradient ascent to improve the value function $Q^{\pi }\left(s_{t},a_{t}|w\right)\nabla_{\psi }\pi \left(s_{t}|\psi \right)$. We use the *LTE I/Q* dataset to train the LSTM to learn a mapping from the sequences of states to the optimal trajectories and from the input features to the objective function and the constraints. The *LTE I/Q* dataset includes sequences of state–action pairs for the UAV [69]. As illustrated in Figure 4, the state includes information about the position, velocity, and environmental conditions of the UAV, while the action represents the trajectory. We use the mean squared error (MSE) loss function to predict future trajectories based on the current state and environmental conditions of the UAV to approximate the behavior of the constraints. We periodically retrain the actor–critic LSTM using updated data to evaluate its accuracy in predicting the UAV trajectory. The actor LSTM predicts trajectory, and the critic updates the parameters based on the predictions. We monitor the convergence of the optimization process and update the LSTM training strategy. The process is repeated throughout the trajectory of the UAV to model the sequential dependencies over time.

### 5.2. Federated Deep Deterministic Policy Gradient (Fed-DDPG)

The fed-DDPG algorithm comprising LSTM in the actor–critic framework aims to enhance the accuracy of individual DDPG algorithms at each agent. This involves exchanging the model parameters among the DDPG agents, allowing the simultaneous optimization of energy consumption for each vehicle and the UAV. The DDPG algorithm is executed on each vehicle, and federated averaging is applied to the parameters obtained from the DDPG algorithm of multiple vehicles to implement the fed-DDPG algorithm. The proposed fed-DDPG algorithm optimizes the agents’ actions together to reduce the entire cost function of the system. An episode is the sequence of events from when the UAV starts flying and either returns to the initial position or the TTI ends. The objective of the UAV is to maximize the average vehicle data traffic and the number of vehicles served. The state at time *t* is the current location of UAV, the current coordinates and the remaining UAV battery power ($P_{t}$). In Figure 4, the UAV at coordinates ($x_{t},y_{t}$) serves vehicles and moves either to the east ($x_{t+1},y_{t}$), south ($x_{t},y_{t-1}$), west ($x_{t-1},y_{t}$), north ($x_{t},y_{t+1}$) or back to the initial point.

### 5.3. Experience Replay and Fed-DDPG

Using experience replay and a target critic network, fed-DDPG enhances the stability and robustness of our proposed solution by minimizing data correlation in various TTIs. Policy exploration and action selection are further improved by adding Gaussian noise to the output of the policy network. In order to model the prior distribution of the UAV height as the UAV trajectory adapts to vehicles’ spatial distribution and traffic demands, we use multivariate Gaussian distribution as per Equations (25) and (26):

(25) $$f_{i}\left(H_{t}\right)∼G\left(\mu_{i}\left(H_{t}\right),v_{i}\left(H_{t}\right)\right)$$

where $\mu_{i}\left(H_{t}\right)$ is the mean vector, and $v_{i}\left(H_{t}\right)$ is the covariance matrix for each sampling value on the trajectory point, without any prior information:

(26) $$f_{i}\left(H_{t}\right)|D_{i}\left(t\right)∼P\left(D_{i}\left(t\right)|f_{i}\left(H_{t}\right)\right)P\left(f_{i}\left(H_{t}\right)\right)$$

At height $H_{t}$, given a maximum tolerable delay, the elements of the covariance matrix in Equation (27) are

(27) $$v_{\tau ,\tau^{\prime }}\left(H_{t}\right)=\exp(-\frac{1}{2}\left|\right|q_{i}\left(\tau \right)-q_{i}\left(\tau^{\prime }\right){\left|\right|}^{2})$$

which implies a larger correlation when two trajectory points are closer to each other. To collect more data from the vehicles, the UAV selects the next trajectory point to maximize the expected value function (V(ψ)). The expected improvement in the value function as the UAV moves to the next set of coordinates in a time slot is based on the constraints C12 and C13 that limit the throughput of the vehicles’ local models. As per Equation (28), the UAV selects an action to move to the next set of coordinates that maximizes the expected delay minimization:

(28) $$a_{i,t}\left(q_{i}\right)=E\left[\max\left\{0,f_{i}\left(q_{i}\right)-f_{i}^{*}\left(q_{i-1}\left(t\right)\right)\right\}\right]$$

where E denotes the expected maximum value function when UAV visited the past coordinates. The next optimal trajectory coordinate in the next TTI is found by maximizing the expected improvement. The UAV periodically updates its location, buffer size, energy status, and channel conditions. The UAV status information adapts the vehicles’ transmission strategies with the UAV trajectories and a vehicle successfully transmits its data to the UAV when the received SNR meets a minimum threshold.

### 5.4. Reward Function

**Figure 5:**
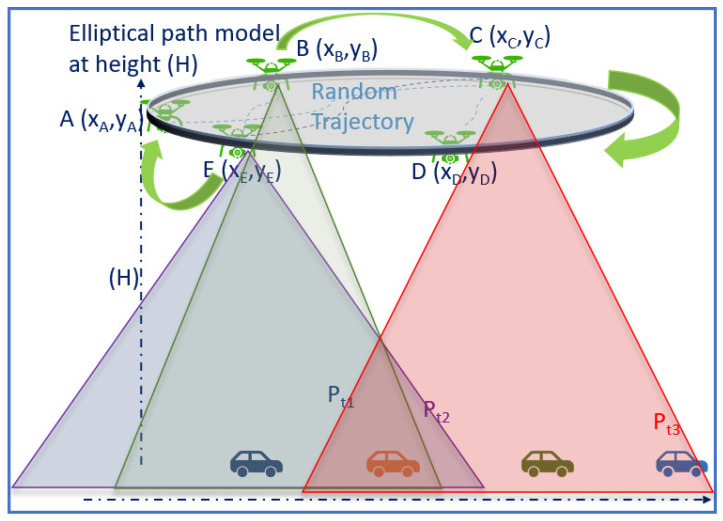
UAV trajectory and vehicle coverage depending on UAV transmit power ($P_{i}\left(t\right)$) and altitude (H). The shaded triangular region ($P_{i}\left(t\right)$) indicates the coverage range of the UAV when the UAV is at a specific altitude (H).

We consider the occupancy of the UAV processor queue as a cost function to define the UAV overall reward function ($r_{i}\left(s_{t},a_{t}\right)$) in each TTI as follows in Equation (29):

(29) $$r_{i}\left(s_{t},a_{t}\right)=\gamma_{1}r_{i,\psi }\left(t\right)+\gamma_{2}r_{i,D}\left(t\right)+\gamma_{3}r_{i,s}\left(t\right)+\gamma_{4}r_{i,P}\left(t\right)$$

where γ is a discount factor, $r_{i,\psi }\left(t\right)$ is the reward that achieves optimal model transmission from the vehicles to the UAV. The term $r_{i,D}\left(t\right)$ is the reward that achieves minimal delay. The term $r_{i,s}\left(t\right)$ is the reward that achieves the maximum data rates, and $r_{i,P}\left(t\right)$ is the reward that achieves the maximum power utilization for UAV. The expectation (E) is taken over all samples in the experience replay buffer and constitutes the global reward $r_{i}\left(s_{t},a_{t}\right)$. Based on the past UAV trajectories, the actor–critic LSTM provides model-free prediction based on the existing samples for action exploration. Consequently, it guides the UAV trajectory towards a more rewarding policy. In fed-DDPG, each vehicle learns a local model based on its data samples and uploads the model to the UAV to maximize the long-term discounted reward in Equation (30):

(30) $$r_{\pi_{i}}=E_{s,a∼E}\left[\sum_{t=0}^{\infty }\gamma_{t}r_{i,t}\left(s,a_{1,t},\ldots ,a_{i,t},\ldots ,a_{I,t}\right)\right]$$

where $a_{I,t}$ is the UAV action in each TTI. When trained on the *LTE I/Q* dataset, the LSTM estimates an optimal coordinate in the next TTI based on the recent trajectory. The action estimation is input to the critic LSTM together with the action learned by the actor LSTM. The critic LSTM evaluates the two actions, and the critic decides an action to be executed. The UAV selects rewarding action with a higher probability to reduce the action space and improve the learning efficiency to map each coordinate to the data size collected from the vehicles to minimize the MSE. The UAV acts as an independent DRL agent, where the policy parameter outputs its action using the deterministic policy based on its observation of the channel states, where *s* is the channel state, *a* is the action of UAV, and E refers to the expectation in the C-V2X environment. The gradient is calculated as per Equation (31) as

(31) $$\nabla_{\phi_{i}}r\left(\pi_{i}\right)=E_{s,a∼E}\nabla_{\phi_{i}}\log\left(\pi_{\phi_{i}}\left(a_{i}|s_{i}\right)\right)+e\left(s_{i},a_{i}\right)$$

where $e\left(s_{i},a_{i}\right)$ is the entropy. The trade-off between maximizing entropy and reward is determined by Equation (32):

(32) $$Q_{i}^{\psi }\left(s,a\right)\leftarrow Q_{i}^{\psi }\left(s,a\right)+\beta \left[r+\gamma \max_{a}Q_{i}^{\psi }\left(s,a^{\prime }\right)-Q_{i}^{\psi }\left(s,a\right)\right]$$

where $Q_{i}^{\psi }$ is the actor–critic Q-function, β∈ (0,1] is the learning rate, and *r* denotes the reward. As illustrated in Figure 5, we assume a random trajectory as well as an elliptical trajectory to model the UAV path. The elliptical trajectory leads to less MSE compared to the random trajectory, as the future coordinates can be accurately predicted. As per Equations (33) and (34), the reward is recursively updated, and the updated reward is used to calculate the loss function using MSE.

(33) $$r_{i}=r_{i-1}+\gamma E_{a^{\prime }∼\pi_{\phi^{\prime }}\left(s^{\prime }\right)}\left[Q_{i}^{\psi^{\prime }}\left(s,a\right)+e\left(s_{i},a_{i}\right)\right]$$

(34) $$\min f_{L_{Q}}\left(\psi \right)=\sum_{i=1}^{I}E_{\left(s,a,r,s^{\prime }\right)∼E}\left[{\left(Q_{i}^{\psi }\left(s,a\right)-r_{i}\right)}^{2}\right]$$

Based on the available battery power ($P_{b}$), the UAV adjusts its height (H). For each height, the number of vehicles covered is depicted by a triangle in Figure 5.

## 6. Simulation Results and Discussion

The vehicles and UAV are trained using a random 10% slice of *V2X-Sim* dataset, split into 60% training data and 40% testing data, to generate sensor data and learn local models [70]. The length and width of the road segment are 300 m by 20 m and the vehicle speed varies between 10 and 100 km/h. The interference experienced by each vehicle is proportional to the relative velocity between the vehicle and the UAV. The threshold for the maximum UAV altitude is (H)+1 km, the noise power is set to −10 dBm, and the BER tolerance is $10^{-4}$. The size of the vehicle data and UAV data for each task is distributed randomly between 1–10 MB and 1–3 MB. Note, the batch size of the local models equals that of the gross data offloading. The transmission and reception powers of the UAV are set to 120 mW and 60 mW, respectively, and the channel bandwidth is set to 20 MHz. We implement the proposed model using *Python* and utilize the *Amazon EC-2* instance to process the datasets. The simulation experiments are executed 50 times for 100–2000 iterations, and the average parameter values are calculated. Table 4 lists the main parameters used in the simulations. Next, we discuss the variation in UAV power ($P_{i}\left(t\right)$) with respect to channel uncertainty caused by the number of vehicles (*V*), UAV trajectory ($q_{\left(x,y\right)}$), and UAV height (H).

### 6.1. Variation in Average Cost Function (UAV Energy and Latency) with Number of Vehicles (*V*)

**Figure 6:**
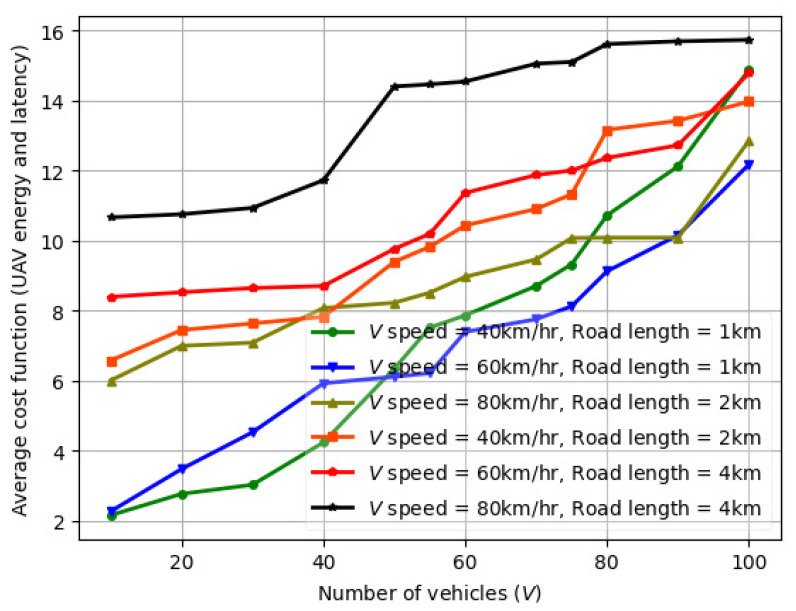
Variation in average cost function (UAV energy and latency) with number of vehicles (*V*).

The variation in average cost function (UAV energy and latency) vs. number of vehicles (*V*) for FL is illustrated in Figure 6 for V=1–100. The average throughput and the average transmission power also increases with the vehicle speed and road segment length. Also, the FL algorithm on vehicles converges after 20 iterations. When vehicles follow FL at a low vehicle speed of V=40 km/h and for a smaller road segment length 1 km, the UAV transmits at a lower power but experiences high interference and reduced throughput. For a higher vehicle speed of V=80 km/h and a larger road segment length 4 km, the UAV power consumption is notably higher.

**Figure 7:**
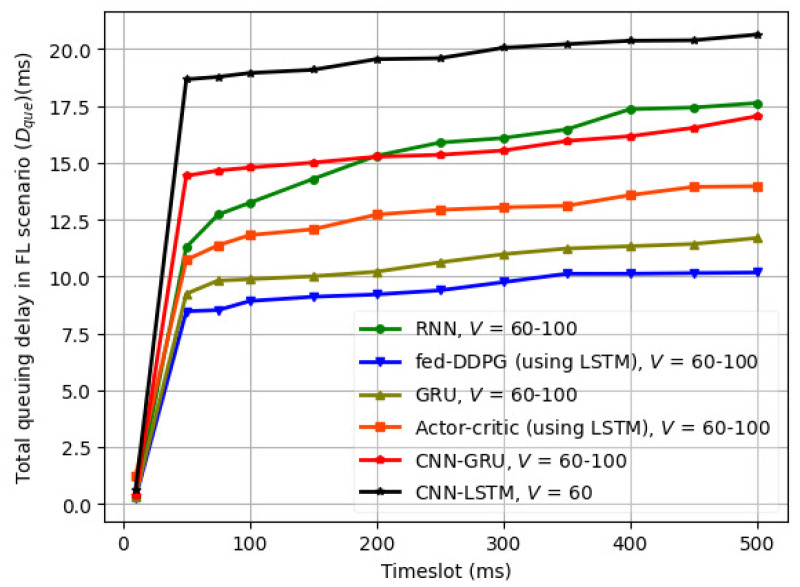
Variation in queuing delay ($D_{que}$) in FL scenario with time slots.

The variation in queuing delay ($D_{que}$) in the FL scenario with time slots is illustrated in Figure 7, where the lowest queuing delay ($D_{que}$) of approximately 11.5 ms is obtained for the fed-DDPG approach. The actor–critic mechanism with LSTM also performs comparably well, with a maximum $D_{que}$ of approximately 13.5 ms. The GRU $D_{que}$ reaches a maximum of approximately 12 ms. As CNN is more suited to image processing applications, the CNN-LSTM and CNN-GRU lead to a higher $D_{que}$ of 15–20 ms.

**Figure 8:**
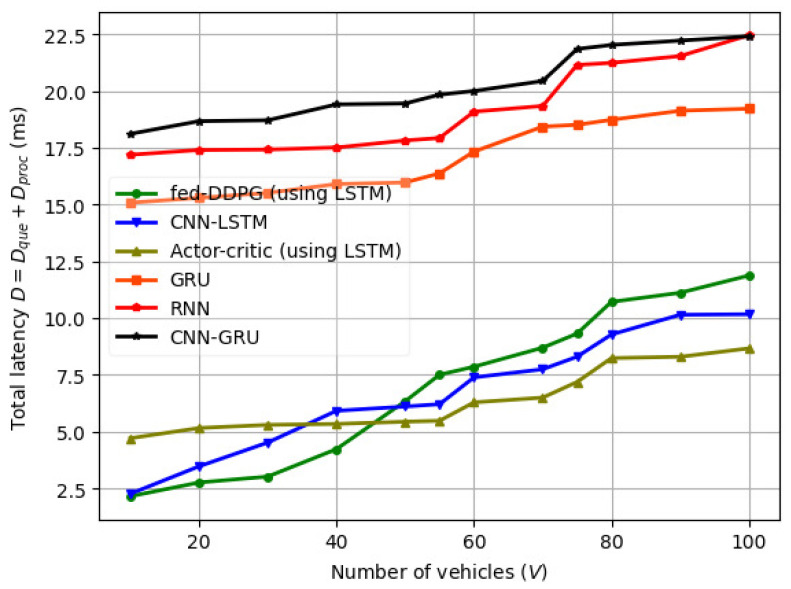
Total delay (D) vs. number of vehicles (*V*) for different machine learning models.

The variation in total delay ($D=D_{que}+D_{proc}$) is illustrated in Figure 8. The variation in D is dependent on the inter-arrival time of the packets. With fed-DDPG, we notice a max D of 13 ms, which is approximately 40% less than the max D of the RNN and CNN-GRU models.

### 6.2. Variation in Average Packet Drop Rate with Control Parameter (ϱ) Using Fed-DDPG

**Figure 9:**
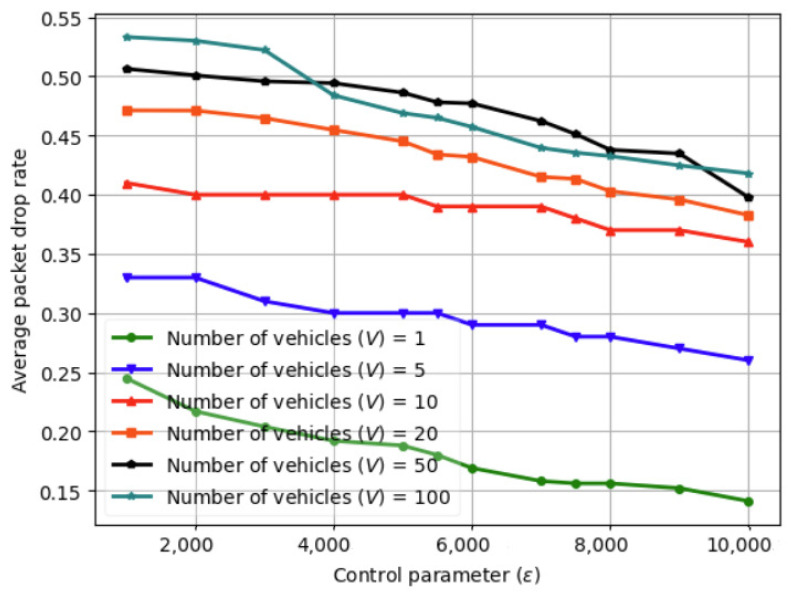
Variation in average packet drop rate with control parameter (ϱ) using fed-DDPG.

The variation in the average packet drop rate with control parameter (ϱ) using fed-DDPG is illustrated in Figure 9 for V=1–100. As we increase ϱ, the model convergence improves, and the average packet drop rate gradually reduces as ϱ is increased from 2000 to 10,000. Increasing ϱ beyond 10,000 increases the required number of iterations and hence increases the latency. Moreover, the transmit power of UAV increases with the increase in the number of vehicles and with the increase in the UAV trajectory fluctuations. In the Rician channel model, the channel gain has a non-monotonic relationship with the flying altitude of the UAV. While increasing the flying altitude improves the channel conditions for certain vehicles and leads to improved throughput, it simultaneously requires higher transmission power to meet the QoS requirement. The results reveal that as ϱ increases, the proposed solution optimizes the task execution time to reduce the queue backlog to ensure queue stability. As the value of ϱ decreases, the priority of the queue backlog increases with the reduced computation rate.

**Figure 10:**
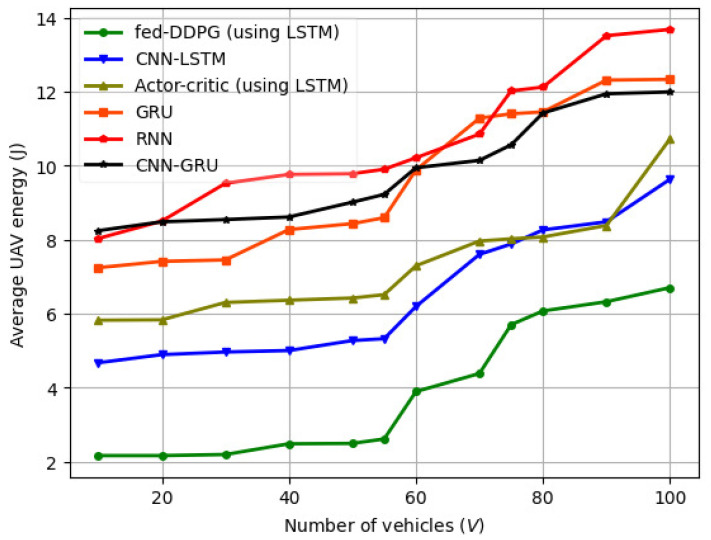
Variation in average UAV energy with number of vehicles (*V*) for different machine learning models.

The variation in the average UAV energy with the number of vehicles at a specific UAV altitude (H) for different packet transmission sizes is illustrated in Figure 10 for the FL model transmission, BSM, and CPM packets. Here, we vary the packet size from 1 to 5 MB. NLoS propagation conditions and path losses are increased in dense urban environments due to obstacles such as buildings, trees, and other structures that follow a Rayleigh distribution. For vehicles to achieve their target SINR thresholds in dense urban areas, the UAV needs to fly at higher altitudes and transmit data at a higher power.

### 6.3. Variation in FL Computation Rate and Average UAV Energy with *V* for Different Machine Learning Models

Here, we consider the effect of wind in the direction of UAV hovering, as well as in the direction against UAV hovering. We add a random SNR to $q_{\left(x,y\right)}$ directions of the UAV’s position, and H, respectively. We execute this iteration for a set of changes in the number of layers and the number of hidden nodes in LSTM. It is observed that when the number of hidden nodes exceeds 32, the average MSE value increases significantly, so it is not necessary to have more than 32 hidden nodes. For scenarios with 1–100 vehicles, due to the limited available resources, not all vehicles achieve their required SINR. Hence, the UAV increases its flying altitude and hence power consumption to provide service to these vehicles to reduce packet drops and retransmissions.

**Figure 11:**
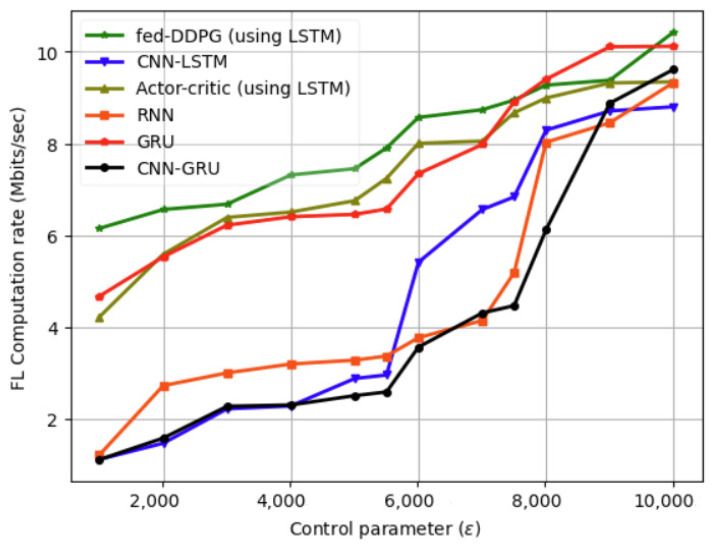
Variation in FL computation rate (Mbits/s) with control parameter (ϱ) for different machine learning models.

As a result, the UAV consumes more energy and has a shorter flight time since it starts from the coordinate origin and does not return to its starting point when it finishes the coverage task. Moreover, when the vehicle data size is smaller, we achieve a higher computation rate for the fed-DDPG algorithm. However, when the vehicle data size increases, the transmission task is completed in a shorter amount of time, and the performance gain increases. Increasing the number of vehicles increases the average UAV power consumption, as the bandwidth available to each vehicle decreases. The variation in FL computation rate (Mbits/s) with control parameter (ϱ) for different machine learning models is illustrated in Figure 11.

### 6.4. Probability of Optimal Trajectory Prediction

**Figure 12:**
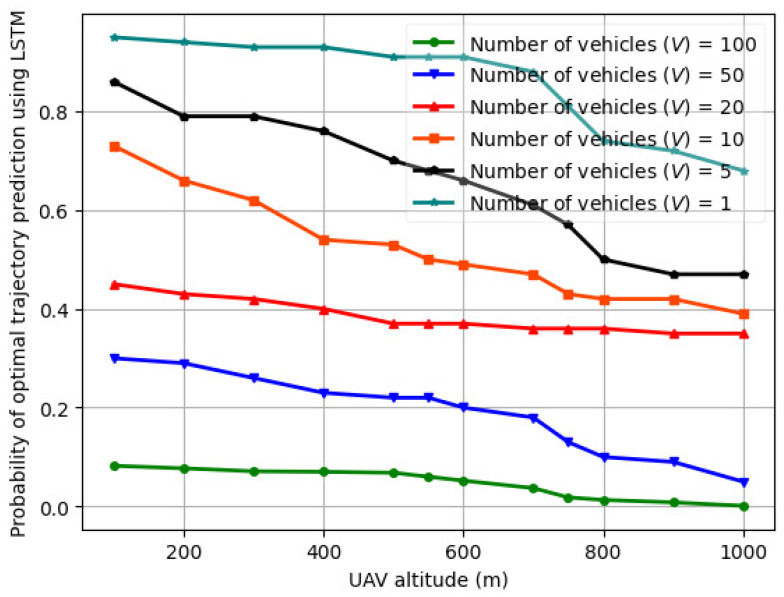
Probability of optimal trajectory prediction for fed-DDPG (using LSTM) vs. UAV altitude (H) for varying number of vehicles (*V*) over trials of 250 episodes.

The variation in the probability of optimal trajectory prediction using LSTM vs. UAV altitude (H) for varying number of vehicles (*V*) is illustrated in Figure 12 for *V* = 1–100. In order to predict a sequence of trajectory coordinates, the LSTM uses past positions or features that represent the movement of an UAV, and predicts the future path. As seen from Figure 12, the probability of optimal trajectory prediction decreases with a greater number of vehicles on the road segment. Also, when the UAV altitude is between 1 and 600 m, the probability of optimal trajectory prediction reduces gradually with the altitude. However, when the UAV altitude is between 600 and 1000 m, the probability of optimal trajectory prediction reduces steeply as compared to an altitude between 1 and 600 m. This is attributed to the available battery power, which restricts UAV movement as well as the number of vehicles the UAV can serve with the available SNR. By combining multiple vehicles, the LSTM improves the sum throughput and reduces the trajectory length, thereby improving the trajectory prediction probability.

**Figure 13:**
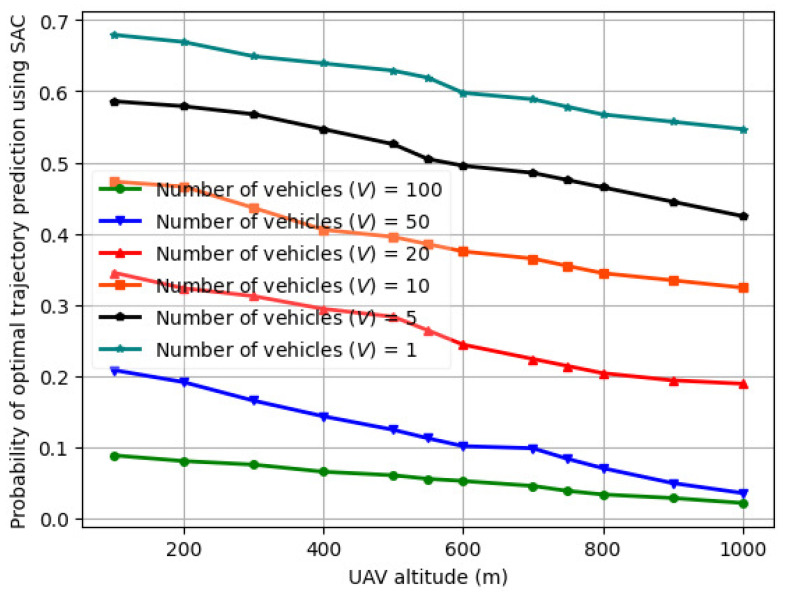
Probability of optimal trajectory prediction for actor–critic (using LSTM) vs. UAV altitude (H) for varying number of vehicles (*V*) over trials of 500 episodes.

**Figure 14:**
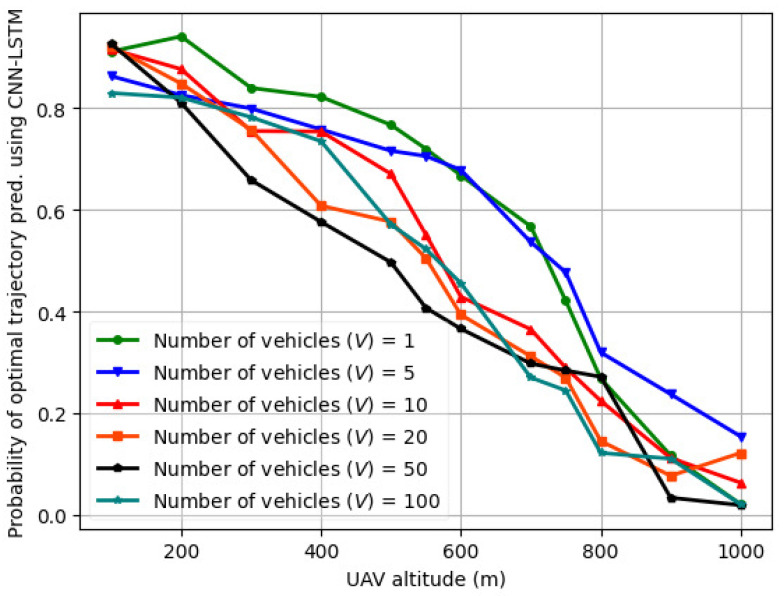
Probability of optimal trajectory prediction for CNN-LSTM vs. UAV altitude (H) for varying number of vehicles (*V*) over trials of 1000 episodes.

**Figure 15:**
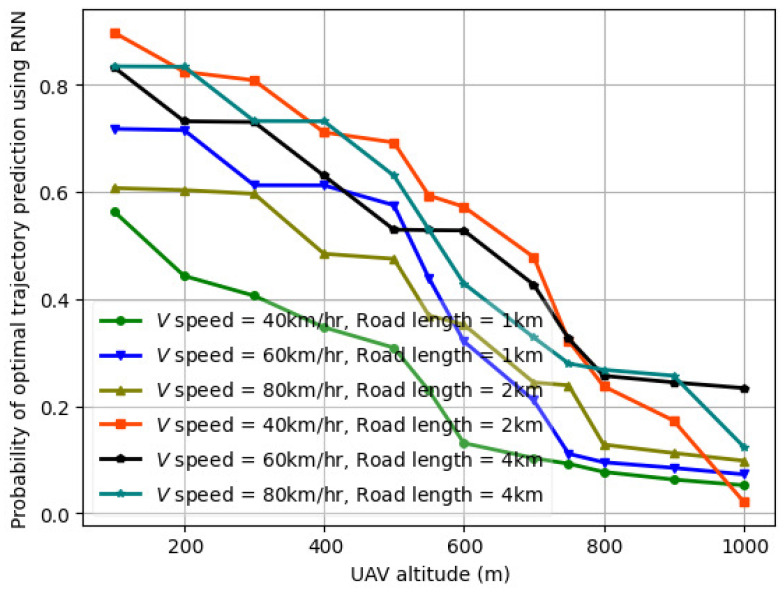
Probability of optimal trajectory prediction for RNN vs. UAV altitude (H) for varying number of vehicles (*V*) over trials of 1000 episodes.

**Figure 16:**
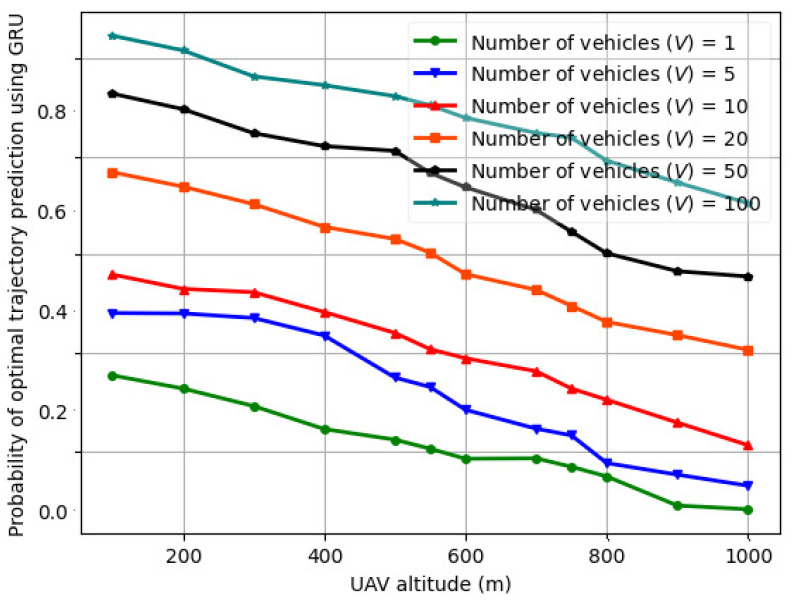
Probability of optimal trajectory prediction for GRU vs. UAV altitude (H) for varying number of vehicles (*V*) over trials of 1000 episodes.

The variation in the probability of optimal trajectory prediction using actor critic vs. UAV altitude (H) for a varying number of vehicles (*V*) is illustrated in Figure 13 for *V* = 1–100. The actor–critic comprises an LSTM in both the actor and critic layers. Compared to Figure 12 for *V* = 1–100, the probability of optimal trajectory prediction using actor–critic is lower than that of fed-DDPG using LSTM for the same number of vehicles, UAV transmit power, road segment length, and UAV altitude. This is because when trained on the *LTE I/Q* dataset for five different UAV altitudes, the fed-DDPG using the LSTM architecture is able to capture complex dependencies in the UAV trajectory samples, and the data processing time is significantly reduced. In the case of actor–critic, when UAV transmits power between 10 and 28 dBm, the trajectory length and sum throughput performance are affected by the number of vehicles and the vehicle speed. This results in slower convergence speed and hence lower probability of optimal trajectory prediction. The probability of optimal trajectory prediction using CNN-LSTM, RNN, and GRU is illustrated in Figure 14, Figure 15, and Figure 16, respectively. As seen, the optimal trajectory prediction declines steeply as compared to fed-DDPG. Also, the CNN-LSTM, RNN, and GRU models are trained for 1000 episodes, whereas actor–critic and fed-DDPG yield a higher probability in 500 and 250 training episodes, respectively.

### 6.5. UAV Transmit Power ($P_{i}\left(t\right)$) vs. SNR for OTFS Modulation

**Figure 17:**
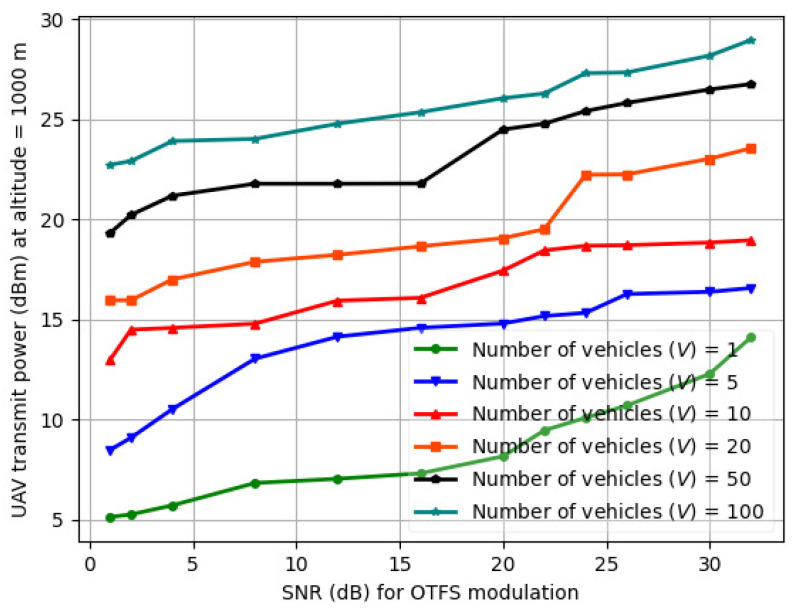
UAV transmit power ($P_{i}\left(t\right)$) vs. SNR in OTFS modulation scheme for varying number of vehicles (*V*).

The variation in UAV transmit power ($P_{i}\left(t\right)$) vs. SNR for OTFS modulation is illustrated in Figure 17 for *V* = 1–100. Low SNR and a larger transmission delay lead to a longer flying time and higher energy consumption. The high mobility of vehicles results in lower SNR as velocity increases, increasing the average power consumption. Low SNR results in decreased system throughput and higher power consumption from UAV to maintain the SNR requirement for vehicles. Higher altitude requires high transmission power in order to meet the SNR requirement for vehicles, which includes both the flying and transmission power of the UAV. The BER is compared at different UAV and vehicle velocities. As the flight speed of the UAV increases, the BER increases, indicating that the Doppler shift from high-speed movement affects communication. It is noted that the difference in BER becomes larger as the SNR increases, and OTFS allows the UAV to communicate over longer distances. With increasing packet size, the weighted sum energy consumption increases, as the UAV is limited in resources to process the incoming packets.

### 6.6. Discussion and Comparison with Existing Works

As shown in Table 5, compared with the previous machine learning approaches whose result is reported as 50% reduction in model convergence time, our method achieves around 30% reduction in latency for similar model convergence time. Furthermore, when the average UAV transmit power is between 1 and 10 dBm, the average task processing time of the gross data offloading and the FL scenarios varies from 5 to 22 ms, respectively. With the increase in packet size for a higher vehicle speed of *V* = 80 km/h and larger road segment length 4 km, the average UAV transmit power is between 10 and 28 dBm.

In this case, the average task processing time of the gross data offloading and the FL scenarios varies from 20 to 35 ms, respectively. When the UAV altitude increases from 600 to 1000 m, the average UAV transmit power varies between 15 and 35 dBm. The flying time is constrained by the average task processing time and number of vehicles in a road segment, respectively. For FL scenario, when the UAV transmit power is above 20 dBm, the processing and queuing delay becomes negligible as compared to the gross data offloading, as part of computation is performed at the edge nodes in the FL scenario. When the average UAV transmit power is higher than 10 dBm, and when the SNR is low, the data offloaded by the vehicles are queued. The average task processing time is determined by its local computation time in the case of FL vs. gross data offloading.

## 7. Conclusions

In this paper, a trajectory optimization mechanism is proposed for UAV-assisted C-V2X communication that considers the Doppler effect caused by high-speed UAV and vehicle motion. Our findings demonstrate that jointly optimizing a vehicle’s transmission time interval, UAV transmit power, and UAV trajectory reduces the weighted total energy consumption of the UAV. Based on past UAV trajectory, a fed-DDPG algorithm is proposed that allows the UAV to estimate a rewarding action by optimizing the trade-off between the maximum flying altitude, power consumption, and number of vehicles served. We formulate the above problem as a mixed-integer non-convex optimization problem and divide the problem into four optimization sub-problems. We utilize an actor–critic framework in fed-DDPG approach, where the actor–critic network comprises an LSTM to estimate and explore the optimal trajectory and altitude for the UAV. Depending on the vehicle’s data distribution and traffic demand, the UAV trajectory is adapted based on the UAV energy consumption and buffer size. Compared to exhaustive search methods that have a high computational complexity, our algorithm reaches a globally optimal solution in polynomial time. Simulation results reveal that the OTFS-based fed-DDPG algorithm is superior to heuristic search algorithms in terms of throughput maximization and latency minimization.

## Figures and Tables

**Table 1:** List of the abbreviations used in this manuscript.

Symbol	Definition
3GPP	Third generation partnership project
5G	Fifth generation (communication networks)
6G	Sixth generation (communication networks)
AoI	Age of information
BER	Bit error rate
BS	Base station
BSM	Basic safety messages
CNN	Convolutional neural network
CPM	Co-operative perception messages
C-V2X	Cellular vehicle-to-everything
DQN	Deep Q-network
DRL	Deep reinforcement learning
FDRL	Federated deep reinforcement learning
FedAvg	Federated averaging
FedSGD	Federated stochastic gradient descent
FL	Federated learning
FL-DDPG	FL-based dual deterministic policy gradient
FRL	Federated reinforcement learning
GNSS	Global navigation satellite system
GRU	Gated recurrent unit
i.i.d.	independent-and-identically-distributed
ITU-R	International Telecommunication Union
LoS	Line of sight
LSTM	long short-term memory
MADRL	Multi-agent deep reinforcement learning
MDP	Markov decision process
MEC	Mobile edge computing
ML	Machine learning
MSE	Mean square error
NLoS	Non-line of sight
NOMA	Non-orthogonal multiple access
NTNs	Non-terrestrial networks
OFDM	Orthogonal frequency division multiplexing
OFDMA	Orthogonal frequency division multiple access
OTFS	Orthogonal time–frequency space
QoS	Quality of service
RL	Reinforcement learning
RNN	Recurrent neural networks
RSSI	Received signal strength indicator
SCA	Successive convex approximation
SINR	Signal-to-interference-plus-noise ratio
SPS	Sensing-based semi-persistent scheduling
SNR	Signal-to-noise ratio
SQP	Successive quadratic programming
TDD	Time-delay-doppler
TTI	Transmission time interval
UAV	Unmanned aerial vehicle

**Table 2:** Advantages of federated reinforcement learning in latency minimization and power optimization of UAV-assisted C-V2X networks.

Benefit	Short Description
Collaborative learning at the vehicles and the UAV	Co-operative learning enables collaboration between multiple vehicle clusters and edge servers for enhanced intelligence [61]. Furthermore, FRL can be utilized to optimize UAV trajectories and data transmission strategies, adapt UAV mobility, optimize computing resources, and make optimal scheduling decisions.
Distributed learning at the vehicles and the UAV	The existing works have demonstrated that FL enhances intelligence and learning across distributed nodes and edge servers. FL has been used in jointly optimizing the UAV flying speed, flying locations, and bandwidth allocation for efficient data collection [62].
Decentralized learning at the vehicles and the UAV	In FL, the vehicles and the vehicle–UAV server pairs train their own models without a centralized aggregator. The vehicles usually collect sensing information, and the timely processing of the sensed information and transmission to the UAV using actor–critic DRL approaches minimizes the AoI by adapting the UAV scheduling strategies [63].
Data availability, reliability, and network scalability; energy-optimal computation offloading	FL allows to leverage the driving decisions obtained from massive amounts of vehicular data, without locally hosting the data [64]. Hence, using FL, new vehicles can easily be integrated into existing clusters to maximize the uplink and downlink capacity of all vehicles [65].
Fault recovery and minimal packet drops	In FL, the global model aggregation is based on local data from multiple vehicles in a cluster. Consequently, it is feasible to rapidly recover the losses due to vehicles experiencing connectivity issues and interference, or if a vehicle’s sensors malfunction. Further, multi-agent FL is used to minimize the UAV flight time by learning the UAV–vehicle association strategies [66].
Communication efficiency and shorter timeouts	Using FL, the model updates and hyper-parameters are communicated to the UAV server rather than gross data offloading. Using asynchronous FL, vehicles can be trained to drop redundant packets. Short-packet transmission can be harnessed to maximize computation efficiency, jointly optimize communication scheduling, and mitigate the impact of intermittent coverage [67].

**Table 3:** Definition of some key symbols and parameters used in this paper.

Symbol	Definition
T	Time period
$P_{b}$	UAV has available battery power
H	UAV flying at a height in meters (m)
$L_{w}$	Transmission windows
V	Number of vehicles in a cluster C
$s_{i,t}$	Data rate in bits/Hz transmitted by each vehicle over UAV flight time
$\psi_{t}$	Binary variable indicating if a vehicle transmits to the UAV in a transmission window
$D_{t}$	Delay which is the sum of the queuing delay and processing delay
$B_{j}$	Bandwidth
$P_{i}^{t}$	Power consumed by the UAV while communicating with the *i*th vehicle
$\sigma^{2}$	Noise power density at the receiver of the vehicle
$h_{i,j}\left[t\right]$	Channel gains of UAV at time slot *t*
$s_{j}^{i}\left(b_{j}^{i},x_{i},y_{i}\right)$	Instantaneous transmission rate for the *i*th vehicle in a time slot
$b_{j}^{i}$	Instantaneous bandwidth utilized by *i*th vehicle in *j*th transmission window
Γ	SNR
$\lambda_{t}$	Inter-arrival time between successive packets
$\Psi_{j}$	Local models considered for federated averaging
$P_{total}\left(t\right)$	Total power consumption during time period *t*
$P_{UAV}^{\left(V\right)}$	Power required for operating the UAV during time period *t*
$P_{i}\left(t\right)$	Power consumption in the *i*th time slot
$\psi_{j}^{i}$	Local models arrive at the queue which accumulates at a rate
$\tau_{u}$	Uplink transmission window
$u_{i}\left(t\right)$	Binary variable; 1 indicates UAV is processing data from a vehicle; 0 indicates UAV is idle
$\psi_{i,k}\left(t\right)$	Size of the *k*th type of vehicular data served by the UAV in the *i*th time slot
$\gamma_{k}\psi_{i,k}\left(t\right)$	Coefficient of power consumption during uplink and downlink of *k*th type of vehicular data
$q_{\left(x,y\right)}$	UAV trajectory
$v_{max}$	Maximum speed of UAV in meter/second (m/s)
${\left(\delta_{t}\right)}^{2}$	Limiting parameter for UAV distance traveled in a TTI
${\hat{h}}_{j}^{i}$	Channel-gain experienced by the *i*th vehicle in *j*th window considering Rician K-factor
$w_{1}-w_{4}$	Weight values scaling factor
$d_{veh}^{uav}$	Distance between the UAV and the *i*th vehicle
$D_{max}$	Upper bound on delay
ψ	Size of packets
ς	Assignment coefficient; 1 denotes vehicle scheduled for transmission, 0 denotes waiting
$\varrho_{i}$	Control parameter for the data rates to reduce the computational complexity of the solution
$Q^{\pi }(s_{t},\pi \left(s_{t}|\psi \right)$	Policy function
V(ψ)	Value functions
$\mu_{i}\left(H_{t}\right)$	Mean vector
$v_{i}\left(H_{t}\right)$	Covariance matrix for each sampling value on the trajectory point
$a_{i,t}\left(q_{i}\right)$	UAV selects action to move to the next coordinates; maximizes expected delay minimization
E	Expected maximum value function when UAV visited the past coordinates
$r_{i}\left(s_{t},a_{t}\right)$	UAV overall reward function
γ	Discount factor
$r_{i,\psi }\left(t\right)$	Reward that achieves optimal model transmission from vehicles to UAV
$a_{I,t}$	UAV action in each TTI
$e\left(s_{i},a_{i}\right)$	Entropy
$Q_{i}^{\psi }$	Actor–critic Q-function
β ∈(0,1]	Learning rate

**Table 4:** Simulation parameters.

Parameter	Value
Vehicle Mobility	Manhattan Mobility
Number of vehicles (*V*)	1–100
Number of UAV	1
UAV deployment altitude	100 m–3 km
Edge server location	In-vehicle
Communication frequency	5.9 GHz
Distance between vehicles	30–100 m
Road length	1–5 km
Vehicle speed	0–100 km/h
Payload size for BSM, CPM	1 byte–3 Megabytes
Payload size of FL models	1 byte–10 Megabytes
Dataset used	V2X-Sim, LTE I/Q
$T_{BSM}$	100 ms–1000 ms
$T_{CPM}$	100, 200, 300, 500 ms
Packet arrival rate (λ)	1000, 2000 packets/s
Speed of UAV	20–50 km/h
UAV transmission power	20 dBm (100 mW)
UAV receiving threshold	−80 dBm
Vehicle transmission power	25 dBm (316.2 mW)
Standard deviation in speed	10 km/h
Noise power, $N_{o}$	−110 dBm
SNR threshold	3 dB
Size of static obstacles	20 m × 20 m
Channel gain	−30 dBm
BER threshold	$10^{-4}$
Doppler speed	30 km/h

**Table 5:** Comparison of different federated learning and deep learning methods for performance enhancement of UAV–vehicle communications.

Reference	Proposed Method	Objectives	Cost Function	Reported Results
[71]	An alternative mechanism to SPS	Lower packet–drop ratio Priority-based strategy to retransmit dropped packets	Delay Number of messages dropped	Reduced delay
[72]	FL-based network optimization	Minimized FL model training losses and convergence time	Node selection Collaborative learning	56% less model convergence time and 3% accuracy enhancement
[73]	Power optimization Optimal resource allocation Delay minimization	Lesser queuing delay Optimal power consumption	Delay Power consumption	79% reduction in communication overhead and 60% shorter queue length
[74]	Integration of edge–cloud platform Used policy-based optimization	Discrete action space Edge–cloud node differentiation Workflow scheduling efficiency	Power consumption Model convergence time Jobs completed within a deadline	56% less power consumption 46% less convergence time
[75,76]	Dempster–Shafer theory-based reputation updates	Periodic feedback Optimal updates based on partial collaboration between participating nodes	Switching factor Delay Network throughput	More vehicles incorporated in the architecture Densely populated servers incorporated in the architecture
Our Work	Trajectory optimization Fed-DDPG and actor–critic using LSTM	Minimize the number of communication rounds between vehicles and UAV Minimize the transmitted hyper parameters Minimize communication delay	Delay Energy consumption Queue backlog and packet drop rate	10% low-latency communication between vehicles (*V*) and UAV and 15% fast model convergence compared to [72,73,74]

## Data Availability

Data is contained within the article.
